# Developing, implementing and disseminating a core outcome set for neonatal medicine

**DOI:** 10.1136/bmjpo-2017-000048

**Published:** 2017-07-26

**Authors:** James Webbe, Ginny Brunton, Shohaib Ali, James MN Duffy, Neena Modi, Chris Gale

**Affiliations:** 1 Section of Neonatal Medicine, Imperial College London, London, UK; 2 Institute of Education, University College London, London, UK; 3 School of Medicine, Imperial College London, London, UK; 4 Nuffield Department of Primary Care Health Sciences, University of Oxford, Oxford, UK

**Keywords:** core outcome set, modified delphi method, neonatal medicine, systematic review

## Abstract

**Background:**

In high resource settings, 1 in 10 newborn babies require admission to a neonatal unit. Research evaluating neonatal care involves recording and reporting many different outcomes and outcome measures. Such variation limits the usefulness of research as studies cannot be compared or combined. To address these limitations, we aim to develop, disseminate and implement a core outcome set for neonatal medicine.

**Methods:**

A steering group that includes parents and former patients, healthcare professionals and researchers has been formed to guide the development of the core outcome set. We will review neonatal trials systematically to identify previously reported outcomes. Additionally, we will specifically identify outcomes of importance to parents, former patients and healthcare professionals through a systematic review of qualitative studies. Outcomes identified will be entered into an international, multi-perspective eDelphi survey. All key stakeholders will be invited to participate. The Delphi method will encourage individual and group stakeholder consensus to identify a core outcome set. The core outcome set will be mapped to existing, routinely recorded data where these exist.

**Discussion:**

Use of a core set will ensure outcomes of importance to key stakeholders, including former patients and parents, are recorded and reported in a standard fashion in future research. Embedding the core outcome set within future clinical studies will extend the usefulness of research to inform practice, enhance patient care and ultimately improve outcomes. Using routinely recorded electronic data will facilitate implementation with minimal addition burden.

**Trial registration number:**

Core Outcome Measures in Effectiveness Trials (COMET) database: 842 (www.comet-initiative.org/studies/details/842).

**What is already known on this topic?**

- Poor outcome selection is an important cause of research waste.
- Much neonatal practice lacks high-quality evidence to support it, and there is considerable variation in neonatal practice and in the outcomes of neonatal care.
- Core outcome sets (an agreed, standardised group of outcomes to be reported by all trials within a research field) have been developed in other specialties to improve outcome selection for research and to facilitate the generation of high-quality evidence.

**What this study hopes to add?**

- Developing a core outcome set for neonatal medicine will identify which outcomes are most important to all affected by neonatal care.
- Standardised reporting of outcomes in clinical trials will facilitate evidence synthesis in neonatal medicine.
- A neonatal core outcome set will also be used to improve the national registry, audit and benchmarking processes.

## Introduction

One in 10 babies born in high resource settings is admitted to a neonatal unit, and this proportion is increasing.^1^ Care provided can range from a few hours of observation to prolonged periods of intensive care, requiring interventions such as intubation and ventilation, intravenous medications and surgery. Neonatal conditions are a leading cause of childhood mortality and morbidity; approximately 2.7 million babies die within the first month of postnatal life each year.^2^ Preterm birth is increasingly implicated in the pathogenesis of adult non-communicable diseases.^3^ Despite this importance, there is a paucity of evidence to underpin many routine neonatal clinical practices, leading to a wide variation in clinical care and uncertain outcomes.^4 5^

It is rare for neonatal systematic reviews and meta-analyses of clinical trials to result in conclusive recommendations for clinical practice.^6^ This is largely due to the different, often incomparable, outcomes measured in individual trials.^7^ In a clinical trial, an outcome is the recorded effect that an intervention has on a participant; for example, a trial of a new antibiotic could involve collection of a number of different outcomes such as survival (to different points in time), side effects or longer-term neurodevelopment. If studies measure different outcomes, or measure them in differing ways using different outcome measures, then results from these studies cannot be compared or combined. Another concern, shared with many paediatric specialties,^8^ is that selection of outcomes rarely involves parents or former patients. The limitations stemming from multiple, non-comparable outcomes are also seen across neonatal observational research, benchmarking, clinical audit and quality improvement studies.

One solution to improve outcome selection, recording and reporting is the development of a core outcome set.^9^ A core outcome set is a collection of important outcomes identified through robust consensus methods by key stakeholders. The purpose of a core outcome set is to ensure that all research in a specialty involves the recording of standard measures, important to key stakeholders and clinically meaningful, which are reported consistently in publications. In recent years, a number of core outcome sets have been developed in both adult and paediatric medicine.^10–13^ The use of core outcome sets for clinical trials is promoted by journals^14^ and research funders.^15^

We aim to develop a core outcome set for neonatal care that incorporates the views of stakeholders including health professionals, researchers, parents and patients.

## Methods

### Prospective registration

The Core Outcome Measures in Effectiveness Trials (COMET) initiative maintains a registry of core outcome sets. We prospectively registered our core outcome set (registration number 842).^16^ Systematic reviews were prospectively registered with Prospective Register of Systematic Reviews (PROSPERO). The review of outcomes reported in neonatal trials has registration number identification CRD42016042110.^17^ The review of qualitative research has registration number identification CRD42016037874.^18^

### Research ethics review

National Health Service (NHS) Research Ethics Committee (REC) approval is not required (see online supplementary additional file 1).

10.1136/bmjpo-2017-000048.supp1 Supplementary material 1

### Study funding

This study is funded as part of a peer-reviewed Medical Research Council Clinician Scientist Fellowship (MR/N008405/1).

### Steering group

A steering group was formed to guide the development of the core outcome set. The members of the steering group have been selected to represent different disciplines, perspectives and expertise. Steering group members are listed at the end of this manuscript.

Within the steering group, a project management team will be established. The project coordinator (JW) and one other member of the steering group (CG) will be responsible for the day-to-day running of the project. The project management team will manage day-to-day aspects of the study; the wider the steering group will be contacted for key decisions. A majority of steering group members need to respond to form a quorum. A decision will be followed when more than half of the quorum agrees.

### Scope of the core outcome set

At the initial steering group meeting, it was decided that the core outcome set will apply to babies receiving medical care on a neonatal unit, with no limitation by gestational age at birth or illness severity. For this project, care delivered exclusively on labour or postnatal wards or in the community is beyond scope. The core outcome set is intended to apply to clinical trials in neonatal care and, where appropriate, to neonatal observational research, benchmarking, audit and quality improvement.

### Study overview

The study will be divided in two distinct stages:

- Identification of outcomes reported in neonatal clinical trials and qualitative research.
- Use of these identified outcomes to determine a set of core outcomes (a neonatal core outcome set) using consensus methods and involving all relevant stakeholders.

### Stage 1: identification of potential outcomes

A search of the Cochrane and PROSPERO databases (11 November 2016) indicated that no systematic review of neonatal outcomes has been performed. Two systematic reviews will be performed to identify outcomes reported in neonatal clinical trials and outcomes reported in qualitative research. All outcomes will be considered, including both those within the neonatal period and outcomes occurring in later life. Where appropriate, different methodological approaches will be used for the systematic review of clinical trials and for the systematic review of qualitative literature.

#### Systematic review to identify outcomes reported in clinical trials

The aim of this systematic review is to identify outcomes that have been recorded in clinical trials for treatments used in neonatal care. We will search the following databases: Cochrane Central Register of Controlled Trials (CENTRAL), Cumulative Index to Nursing and Allied Health Literature (CINAHL), Excerpta Medica Database (EMBASE) and Medical Literature Analysis and Retrieval System Online (MEDLINE); the proposed search strategy is included (see online supplementary additional file 2). Randomised or cluster randomised trials evaluating interventions for neonates (age 0–28 days) of any gestation or infants requiring ongoing care in a neonatal unit will be included. All trials published in the last 5 years will be included. Translations will be sought if a trial is not reported in English. When screening studies for inclusion, studies will be reviewed by two independent reviewers (SA and JW). Where there is disagreement, the study will be reassessed by an additional reviewer (CG).

10.1136/bmjpo-2017-000048.supp2 Supplementary material 2

Using a pilot-tested extraction form, data will be extracted relating to trial identifiers, number of infants recruited, primary and secondary outcomes, whether the study was prospectively registered and whether there is evidence of parent or patient involvement in outcome selection. Two reviewers will independently assess the methodological quality of the included studies using the Jadad scale (score range 1 to 5).^19^ Methodological quality will be dichotomised as high (score ≥3) and low (score <3). Outcomes will be extracted from included trials; the primary outcome(s) will be defined as the outcome explicitly defined as such or used for a sample size calculation; secondary outcomes will be all other outcomes. Studies will be reviewed by two independent reviewers (SA and JW). Where there is disagreement, the study will be reassessed by an additional reviewer (CG).

Results will be reported in a narrative synthesis and presented in a tabular form. Outcomes will be grouped using a previously defined framework of biological systems.^20^ The number and frequency of outcomes measured in clinical trials will be reported. Additional analyses will be conducted to examine the frequency with which predefined neonatal comorbidities are reported in the largest clinical trials (see online supplementary additional file 3).

10.1136/bmjpo-2017-000048.supp3 Supplementary material 3

#### Systematic review to identify outcomes reported by parents, patients and other stakeholders

The aim of this systematic review is to identify which outcomes of neonatal care are described by former patients, parents and healthcare providers as important.

We will search the following databases: Applied Social Sciences Index and Abstracts, CENTRAL, CINAHL, EMBASE, MEDLINE and Psychological Information Database. The proposed search strategy is included (see online supplementary additional file 4). As this review relates to qualitative research, the review question has been formulated in a SPIDER format to define eligibility criteria.^21^ Qualitative studies (including phenomenology, ethnography, case studies and grounded theory) relating to neonates (age 0–28 days) of any gestation and any infants requiring ongoing care in a neonatal unit beyond this age in a high resource setting will be included. Quantitative research will be included if relevant data have been gathered, for example, surveys developed with parent or caregiver input. All studies published in a peer-reviewed journal or a funder's report in the last 20 years will be included. Non-English trial reports will be translated. The primary outcome will be a comprehensive list of neonatal care outcomes reported by former patients, their parents and the healthcare professionals caring for them. All screening will be done by one individual (JW), but for quality assurance, an independent reviewer (CG) will screen 10% of abstracts and titles. We will examine for agreement using Cohen's Kappa coefficient.

10.1136/bmjpo-2017-000048.supp4 Supplementary material 4

After screening, all papers will be coded using a piloted coding tool and using Eppi-Reviewer 4.^22^ The coding tool will be pilot tested on 10 papers and refined accordingly. All papers will be coded independently by two researchers (JW and CG/GB), with any disagreement resolved by a third researcher (CG/GB). Data will be extracted from each study relating to details of the stakeholders included, the gestational age and birth weight of neonates involved, the country in which the study was performed, the qualitative methodology used and the outcomes identified by stakeholders.

Outcomes identified by multiple studies will be combined. Outcomes identified will be grouped according to a framework of biological health systems.^20^ Textual findings from data related to each organ system will be analysed as part of a thematic synthesis to determine key themes. Further thematic analysis will be undertaken to identify novel outcome categories. Additional analyses will examine whether outcomes differ by stakeholder groups and gestational age.

### Stage 2: determining core outcomes

A comprehensive inventory of outcomes will be created by combining the results of the two systematic reviews. The steering group will eliminate duplicate and group outcomes based on a framework of biological health systems.^20^ These will be used as the starting point for the consensus process to determine a core outcome set. Consensus methods will involve multiple stakeholder groups; two methods will be used, a multi-round, online Delphi survey followed by a consensus meeting.

The following groups of stakeholders will be identified:

- Former patients admitted to a neonatal unit in infancy and parents of neonatal patients. These will be recruited by placing adverts on neonatal charity websites and contacting bloggers discussing preterm and neonatal experiences.
- Clinicians including neonatal nurses, neonatologists, general paediatricians, paediatric specialists and community paediatricians specialising in neurodevelopment. Recruitment will be through adverts placed on the Royal College of Paediatrics and Child Health (RCPCH) website and through professional organisations such as the British Association of Perinatal Medicine and the Neonatal Society.
- Allied health professionals including physiotherapists, speech and language therapists, occupational therapists and clinical psychologists. Adverts will be placed in professional journals and on profession-specific specialty interest websites.
- Academics and researchers in the neonatal field or those involved in the collection of routine neonatal datasets. Recruitment will be through national meetings, academic publications and through academic organisations.

We will aim for 30 or more participants in each group, which will give a total panel of at least 120 participants, in line with previous core outcome methods.^23^

The panel will undergo a three-round Delphi survey (using a web-based questionnaire) to establish consensus. The Delphi process allows for consensus to be reached from a selection of disparate expert opinions.^24^ In each round, panel members will be asked to rank the outcomes. In the first round of the Delphi process, panel members will also have the opportunity to suggest additional outcomes that they feel are important and were not identified in the two systematic reviews. These will be integrated into round 2 by the study management group following principles established by the steering group. The Delphi surveys will enable all stakeholders to participate and will assess the extent of agreement (consensus measurement) and resolve disagreement (consensus development).

Panel members will be asked to score each outcome between 1 and 9 during each round, using a Likert-type scale.^25^ After each round, results will be collated and any outcomes universally scored to be of limited importance (scored between 1 and 3) will not be carried forward to the next round. In later rounds, panel members will be presented with the median score, by stakeholder group, for each outcome and asked to review their results before re-scoring outcomes. Repeated reflection and scoring has been demonstrated to increase the likelihood of convergence and consensus.^26^

In the final round, panel members will also be asked whether each outcome should be included in the core outcome set. A standardised definition will then be applied to the results from this round:

- Consensus in (classified as a core outcome): Over 70% of panel members in each group score the outcome ‘critical for decision making’ (score 7 to 9) and less than 15% of panel members in each group score the outcome of limited importance for decision making’ (score 1 to 3).
- Consensus out (do not classify as a core outcome): Over 70% of panel members in each group score the outcome ‘of limited importance for decision making’ (score 1 to 3) and less than 15% of panel members in each group score the outcome ‘critical for decision making’ (score 7 to 9).
- No Consensus (do not classify as a core outcome): Anything else.

The results of this final round will be taken to a consensus meeting to determine a final core outcome set.

A consensus meeting of stakeholders will use the results of the completed Delphi process to identify a final core outcome set. The remit of the consensus meeting will be to refine the final results from the Delphi; no new outcomes will be considered at this stage and the results of the Delphi will be paramount when selecting the core outcome set. To ensure transparency, these outcomes will be published with the survey results to show the degree of consensus for each outcome. If outcomes are excluded at this stage, they will also be published (with survey results) with an explanation of the reasons for exclusion and which stakeholder groups agreed or disagreed with the exclusion. The core outcome set will be reported in line with reporting guidelines.^27^

## Discussion

Once identified, the core outcome set will be disseminated to raise awareness and uptake. We will publish our methodology and results in peer-reviewed journals. Raising the awareness of the core outcome set among neonatal practitioners and researchers is a crucial part of this project. The core outcome set will be made freely available through the COMET initiative website and through the Core Outcomes in Women's and Newborn Health Network Initiative.^28^ Use of the core outcome set in future research will be promoted by research funders^15^ and journals.^14^ In the UK, a standardised set of data items, the Neonatal Data Set (NDS), exists for admissions to NHS neonatal care. These data form the National Neonatal Research Database (NNRD), a national resource used for multiple purposes including research,^29^ benchmarking and national audit.^30^ We will work with the Neonatal Data Analysis Unit and other bodies to ensure data relating to the core outcomes are extracted from routinely collected data and held in the NNRD. The NDS is reviewed annually and therefore provides an opportunity for neonatal core outcomes to be captured from routinely recorded data. This will facilitate audit and benchmarking with a standardised group of outcomes that are important to former patients, parents and clinicians.

A core outcome set in neonatal medicine will ensure that researchers select outcomes that are clinically relevant and important to parents and patients. Implementing a core outcome set in neonatal medicine will standardise the selection of outcomes in individual clinical trials. A standardised set of outcomes across studies will facilitate evidence synthesis in meta-analyses and systematic reviews. This will assist the delivery of evidence-based improvements in neonatal care.
