# Supplementary material for: Developing, implementing and disseminating a core outcome set for neonatal medicine
Source: BMJ Paediatr Open. 2017 Jul 26;1(1):e000048. doi: 10.1136/bmjpo-2017-000048 (PMC5862188; doi:10.1136/bmjpo-2017-000048)
Supplement: Supplementary material 1 [file bmjpo-2017-000048supp001.pdf]

Go straight to content.

Medical Research Council MRC

NHS Health Research Authority

Do I need NHS REC approval?

To print your result with title and IRAS Project ID please enter your details below:

Title of your research:

Core Outcomes in Neonatology

IRAS Project ID (if available):

Your answers to the following questions indicate that **you do not need NHS REC approval for sites in England.** However, **you may need other approvals.**

You have answered ***'YES'*** to: Is your study research?

You answered ***'NO'*** to all of these questions:

Question Set 1

- Is your study a clinical trial of an investigational medicinal product?
- Is your study one or more of the following: A non-CE marked medical device, or a device which has been modified or is being used outside of its CE mark intended purpose, and the study is conducted by or with the support of the manufacturer or another commercial company (including university spin-out company) to provide data for CE marking purposes?
- Does your study involve exposure to any ionising radiation?
- Does your study involve the processing of disclosable protected information on the Register of the Human Fertilisation and Embryology Authority by researchers, without consent?
- Is your study a clinical trial involving the participation of practising midwives?

Question Set 2

- Will your study involve research participants identified from, or because of their past or present use of services (adult and children's healthcare within the NHS and adult social care), for which the UK health departments are responsible (including services provided under contract with the private or voluntary sectors), including participants recruited through these services as healthy controls?

- Will your research involve collection of tissue or information from any users of these services (adult and children's healthcare within the NHS and adult social care)? This may include users who have died within the last 100 years.
- Will your research involve the use of previously collected tissue or information from which the research team could identify individual past or present users of these services (adult and children's healthcare within the NHS and adult social care), either directly from that tissue or information, or from its combination with other tissue or information likely to come into their possession?
- Will your research involve research participants identified because of their status as relatives or carers of past or present users of these services (adult and children's healthcare within the NHS and adult social care)?

Question Set 3

- Will your research involve the storage of relevant material from the living or deceased on premises in the UK, but not Scotland, without an appropriate licence from the Human Tissue Authority (HTA)? This includes storage of imported material.
- Will your research involve storage or use of relevant material from the living, collected on or after 1st September 2006, and the research is not within the terms of consent from the donors, and the research does not come under another NHS REC approval?
- Will your research involve the analysis of DNA from bodily material, collected on or after 1st September 2006, and this analysis is not within the terms of consent for research from the donor?

Question Set 4

- Will your research involve at any stage intrusive procedures with adults who lack capacity to consent for themselves, including participants retained in study following the loss of capacity?
- Is your research health-related and involving prisoners?
- Does your research involve xenotransplantation?
- Is your research a social care project funded by the Department of Health?

If your research extends beyond **England** find out if you need NHS REC approval by selecting the *'OTHER UK COUNTRIES'* button below.

OTHER UK COUNTRIES

If, after visiting all relevant UK countries, this decision tool suggests that you do not require NHS REC approval follow this link for final confirmation and further information.

Print This Page

NOTE: If using Internet Explorer please use browser print function.

About this tool **Feedback** **Contact** **Glossary**
