# Supplementary material for: Developing, implementing and disseminating a core outcome set for neonatal medicine
Source: BMJ Paediatr Open. 2017 Jul 26;1(1):e000048. doi: 10.1136/bmjpo-2017-000048 (PMC5862188; doi:10.1136/bmjpo-2017-000048)
Supplement: Supplementary material 2 [file bmjpo-2017-000048supp002.pdf]

SEARCH STRATEGY FOR SYSTEMATIC REVIEW OF CLINICAL TRIALS

The following search strategy was utilised to search the CINAHL database:

1. CINAHL; neonat*.ti,ab; 26427 results.
2. CINAHL; newborn*.ti,ab; 12536 results.
3. CINAHL; prematur*.ti,ab; 12204 results.
4. CINAHL; preterm.ti,ab; 12705 results.
5. CINAHL; infan*.ti,ab; 46987 results.
6. CINAHL; neonat*.ti,ab; 26427 results.
7. CINAHL; baby.ti,ab; 48441 results.
8. CINAHL; babies.ti,ab; 46210 results.
9. CINAHL; 3 OR 4; 22678 results.
10. CINAHL; 5 OR 6 OR 7 OR 8; 77600 results.
11. CINAHL; 9 AND 10; 12436 results.
12. CINAHL; NEONATOLOGY/; 495 results.
13. CINAHL; INTENSIVE CARE UNITS, NEONATAL/; 7292 results.
14. CINAHL; INTENSIVE CARE, NEONATAL/; 3031 results.
15. CINAHL; NICU.ti,ab; 3307 results.
16. CINAHL; SCBU.ti,ab; 35 results.
17. CINAHL; 1 OR 2 OR 11 OR 12 OR 13 OR 14 OR 15 OR 16; 44276 results.
18. CINAHL; (treatment AND outcome).ti,ab; 59360 results.
19. CINAHL; OUTCOME ASSESSMENT/ OR TREATMENT OUTCOMES/; 147912 results.
20. CINAHL; (key AND outcome).ti,ab; 8935 results.
21. CINAHL; (outcome AND measure).ti,ab; 54009 results.

22. CINAHL; (core AND outcome).ti,ab; 2512 results.

23. CINAHL; 18 OR 19 OR 20 OR 21 OR 22; 224757 results.

24. CINAHL; (clinical AND trial).ti,ab; 49323 results.

25. CINAHL; RANDOMIZED CONTROLLED TRIALS/ OR CLINICAL TRIALS/; 111053 results.

26. CINAHL; randomized.ti,ab; 68549 results.

27. CINAHL; (single AND blind AND procedure).ti,ab; 178 results.

28. CINAHL; SINGLE-BLIND STUDIES/; 6933 results.

29. CINAHL; (double AND blind AND procedure).ti,ab; 499 results.

30. CINAHL; DOUBLE-BLIND STUDIES/; 22288 results.

31. CINAHL; 24 OR 25 OR 26 OR 27 OR 28 OR 29 OR 30; 177369 results.

32. CINAHL; 17 AND 23 AND 31; 1560 results.

33. CINAHL; 32 [Limit to: Publication Year 1997-2016]; 1532 results.

34. CINAHL; DELIVERY, OBSTETRIC/ OR OBSTETRIC PATIENTS/ [Limit to: Publication Year 1997-2016]; 3894 results.

35. CINAHL; 33 NOT 34 [Limit to: Publication Year 1997-2016]; 1509 results.

The terms derived from this search strategy were translated out into other electronic database sources.
