# Supplementary material for: Developing, implementing and disseminating a core outcome set for neonatal medicine
Source: BMJ Paediatr Open. 2017 Jul 26;1(1):e000048. doi: 10.1136/bmjpo-2017-000048 (PMC5862188; doi:10.1136/bmjpo-2017-000048)
Supplement: Supplementary material 3 [file bmjpo-2017-000048supp003.pdf]

PREDEFINED NEONATAL COMORBIDITIES

Respiratory distress syndrome

Bronchopulmonary dysplasia

Intraventricular haemorrhage

Periventricular leucomalacia

Retinopathy of prematurity

Neurodevelopmental outcome

Patent ductus arteriosus

Necrotising enterocolitis

Time to full enteral feeds

Duration of parenteral feeding

Breastfeeding

Sepsis

Death
