# Supplementary material for: Developing, implementing and disseminating a core outcome set for neonatal medicine
Source: BMJ Paediatr Open. 2017 Jul 26;1(1):e000048. doi: 10.1136/bmjpo-2017-000048 (PMC5862188; doi:10.1136/bmjpo-2017-000048)
Supplement: Supplementary material 4 [file bmjpo-2017-000048supp004.pdf]

SEARCH STRATEGY FOR SYSTEMATIC REVIEW OF QUALITATIVE EVIDENCE

The following search strategy was utilised to search the Medline database:

1. Medline; neonat*.ti,ab;
2. Medline; exp INFANT, NEWBORN/;
3. Medline; ((preterm OR "pre term" OR prematur*) AND (infan* OR neonat* OR baby OR babies)).ti,ab;
4. Medline; (infant* adj2 premature).af;
5. Medline; exp NEONATOLOGY/;
6. Medline; exp INTENSIVE CARE UNITS, NEONATAL/;
7. Medline; exp INTENSIVE CARE, NEONATAL/;
8. Medline; ("intensive care" adj2 neonat*).ti,ab;
9. Medline; NICU.ti,ab;
10. Medline; SCBU.ti,ab;
11. Medline; "special care baby unit*".ti,ab;
12. Medline; INFANT, PREMATURE, DISEASES/;
13. Medline; 1 OR 2 OR 3 OR 4 OR 5 OR 6 OR 7 OR 8 OR 9 OR 10 OR 11 OR 12;
14. Medline; (treatment adj2 outcome*).ti,ab;
15. Medline; exp TREATMENT OUTCOME/;
16. Medline; "key outcome*".ti,ab;
17. Medline; (important adj2 outcome*).ti,ab;
18. Medline; exp PATIENT OUTCOME ASSESSMENT/;
19. Medline; (outcome* adj2 set).ti,ab;
20. Medline; exp "OUTCOME ASSESSMENT (HEALTH CARE)"/;
21. Medline; "outcome assessment*".ti,ab;
22. Medline; (core adj2 outcome*).ti,ab;
23. Medline; "outcome measure*".ti,ab;

24. Medline; 14 OR 15 OR 16 OR 17 OR 18 OR 19 OR 20 OR 21 OR 22 OR 23;

25. Medline; ("semi-structured" OR semistructured OR unstructured OR informal OR "indepth" OR indepth OR "face-to-face").ti,ab;

26. Medline; ((structured OR guide) adj3 (interview* OR discussion*)).ti,ab;

27. Medline; ("focus group*" OR qualitative OR ethnograph* OR fieldwork OR "field work" OR "key informant*").ti,ab;

28. Medline; exp INTERVIEWS AS TOPIC/;

29. Medline; exp FOCUS GROUPS/;

30. Medline; exp NARRATION/;

31. Medline; exp QUALITATIVE RESEARCH/;

32. Medline; 25 O 26 OR 27 OR 28 OR 29 OR 30 OR 31 OR 32;

33. Medline; 13 AND 24 AND 32;

The terms derived from this search strategy were translated out into other electronic database sources.
